# Identification of Molecular Profile of Ear Fibroblasts Derived from Spindle-Transferred Holstein Cattle with Ooplasts from Taiwan Yellow Cattle under Heat Stress

**DOI:** 10.3390/ani14091371

**Published:** 2024-05-02

**Authors:** Yu-Ju Lee, Jai-Wei Lee, Chao-Wei Huang, Kuo-Tai Yang, Shao-Yu Peng, Chi Yu, Yen-Hua Lee, I-Ling Lai, Perng-Chih Shen

**Affiliations:** 1 Graduate Institute of Bioresources, National Pingtung University of Science and Technology, Neipu, Pingtung 91201, Taiwan; p10321005@mail.npust.edu.tw (Y.-J.L.); ilai@mail.npust.edu.tw (I.-L.L.); 2 Department of Tropical Agriculture and International Cooperation, National Pingtung University of Science and Technology, Neipu, Pingtung 91201, Taiwan; joeylee@mail.npust.edu.tw (J.-W.L.); cwhuang@mail.npust.edu.tw (C.-W.H.); 3 Department of Animal Science, National Pingtung University of Science and Technology, Neipu, Pingtung 91201, Taiwan; ktyang@mail.npust.edu.tw (K.-T.Y.); sypeng@mail.npust.edu.tw (S.-Y.P.); chiyu@mail.npust.edu.tw (C.Y.); yhlee@mail.npust.edu.tw (Y.-H.L.)

**Keywords:** heat stress, cytoplasmic origin, apoptotic factors, antioxidant, oxidative phosphorylation, thermotolerance, Holstein, Taiwan yellow cattle

## Abstract

**Simple Summary:**

We previously demonstrated that cells of cows produced from reconstructed embryos containing the cytoplasm of a heat-tolerant breed (*Bos indicus,* BI) showed improved thermotolerance despite their nuclei being derived from a heat-sensitive breed (*Bos Taurus,* BT). In this study, we utilized spindle transfer (ST) technology to transfer karyoplasts from heat-sensitive Holstein cattle (H) into recipient ooplasm from heat-tolerant Taiwan yellow cattle (Y). This process resulted in the creation of ST oocytes (ST-Yo-Hn). These ST oocytes were subsequently subjected to in vitro fertilization (IVF) with Holstein bull semen, resulting in fertilized ST embryos. The resulting ST-Yo-Hn blastocysts were then transferred into recipient females, leading to the successful production of three ST-Yo-Hn cattle. To understand the factors mediating thermotolerance, ear fibroblasts from ST-Yo-Hn and H cattle were isolated, and their differentially expressed protein and gene profiles under heat shock (42 °C for 12 h) were compared. The results indicated that the ear fibroblasts of the ST-Yo-Hn cattle exhibited significantly (*p* < 0.05) lower expression of pro-apoptotic factors, higher expression of anti-apoptotic factors, and higher antioxidant ability under heat stress, making the somatic cells from the ST-Yo-Hn cattle more heat-tolerant than those from the H cattle.

**Abstract:**

Global warming has a significant impact on the dairy farming industry, as heat stress causes reproductive endocrine imbalances and leads to substantial economic losses, particularly in tropical–subtropical regions. The Holstein breed, which is widely used for dairy production, is highly susceptible to heat stress, resulting in a dramatic reduction in milk production during hot seasons. However, previous studies have shown that cells of cows produced from reconstructed embryos containing cytoplasm (o) from Taiwan yellow cattle (Y) have improved thermotolerance despite their nuclei (n) being derived from heat-sensitive Holstein cattle (H). Using spindle transfer (ST) technology, we successfully produced ST-Yo-Hn cattle and proved that the thermotolerance of their ear fibroblasts is similar to that of Y and significantly better than that of H (*p* < 0.05). Despite these findings, the genes and molecules responsible for the different sensitivities of cells derived from ST-Yo-Hn and H cattle have not been extensively investigated. In the present study, ear fibroblasts from ST-Yo-Hn and H cattle were isolated, and differentially expressed protein and gene profiles were compared with or without heat stress (hs) (42 °C for 12 h). The results revealed that the relative protein expression levels of pro-apoptotic factors, including Caspase-3, -8, and -9, in the ear fibroblasts from the ST-Yo-Hn-hs group were significantly lower (*p* < 0.05) than those from the H-hs group. Conversely, the relative expression levels of anti-apoptotic factors, including GNA14 protein and the *CRELD2* and *PRKCQ* genes, were significantly higher (*p* < 0.05) in the ear fibroblasts from the ST-Yo-Hn-hs group compared to those from the H-hs group. Analysis of oxidative phosphorylation-related factors revealed that the relative expression levels of the *GPX1* gene and Complex-I, Complex-IV, CAT, and PGLS proteins were significantly higher (*p* < 0.05) in the ear fibroblasts from the ST-Yo-Hn-hs group compared to those from the H-hs group. Taken together, these findings suggest that ear fibroblasts from ST-Yo-Hn cattle have superior thermotolerance compared to those from H cattle due to their lower expression of pro-apoptotic factors and higher expression of oxidative phosphorylation and antioxidant factors. Moreover, this improved thermotolerance is attributed, at least partially, to the cytoplasm derived from more heat-tolerant Y cattle. Hence, using ST technology to produce more heat-tolerant H cattle containing Y cytoplasm could be a feasible approach to alleviate the negative impacts of heat stress on dairy cattle in tropical–subtropical regions.

## 1. Introduction

Cattle breeds around the world can be classified into two main categories: *Bos indicus* (BI) breeds, which include Brahman, Sahiwal, Tharparkar, Nelore, Boran, Tuli, Gir, Gyr, and Taiwan yellow cattle, and *Bos Taurus* (BT) breeds, which include Holstein, Angus, and Hereford. Under heat stress, Taiwan yellow cattle (Y; BI) have lower body temperatures than Holstein (H; BT) cattle, indicating that BI breeds are more heat-tolerant than BT breeds [1,2,3].

In addition, studies have demonstrated that ear cells derived from Y contribute to higher survival rates than H ear cells under heat stress [3]. Furthermore, in vitro studies have shown that somatic cells derived from BI breeds, such as Brahman, exhibit better survival rates than those derived from BT breeds, such as Angus and Holstein, under heat stress [1,4]. Therefore, it can be concluded that the thermotolerance of BI breeds is better than that of BT breeds.

Despite being sensitive to heat stress, Holstein cattle (BT breed) remain the most widely used dairy breed worldwide. Current climate changes, especially global warming, have become an increasingly noticeable phenomenon [5,6] and are of concern to the dairy industry. In tropical and subtropical regions, heat stress affects the endocrine systems of Holstein cattle [7], leading to reduced pregnancy rates, increased miscarriage rates [8,9], and lower milk production [10,11]. Under these circumstances, it is common practice to crossbreed native heat-tolerant cattle breeds such as Gir cattle (BI breed) with Holstein cattle to produce hybrid dairy cattle that have better heat tolerance and acceptable milk production in many tropical regions [12,13]. Although the milk production of Gir X H hybrid cows is not as high as that of purebred H cows, it is still significantly higher than that of purebred Gir cattle [14] (Santos et al., 2012). Moreover, it has been demonstrated that Sahiwal cattle (BI) have significantly lower rectal temperatures in the hot season compared to Frieswal cattle, a crossbreed of Tharparkar (BI) and H [15]. Obviously, this approach compromises heat tolerance and milk production, which leaves something to be desired.

To understand the role of the nucleus or cytoplasm in the response to heat stress in cattle, somatic cell nuclear transfer (SCNT) and spindle transfer (ST) technologies have been applied to produce reconstructed embryos with nuclei and cytoplasm originating from different bovine breeds. It has been observed that when an oocyte is derived from a heat-tolerant breed (Brahman), the resulting embryos have better heat tolerance, irrespective of whether the sperm is from a heat-sensitive or heat-tolerant breed [16,17]. We previously demonstrated that SCNT embryos derived from ooplasm from H cattle (Ho-Yd (H ooplasm and Y donor) and Ho-Hd (H ooplasm and H donor)) have lower development ability under heat stress compared to those derived from Y cattle (Yo-Yd (Y ooplasm and Y donor) and Yo-Hd (Y ooplasm and H donor)) [18]. In addition, the levels of apoptosis-related proteins, such as Bax; Cytochrome C; and Caspase-3, -8, and -9, are lower in the ear cells of SCNT-Yo-Hd cattle compared to SCNT-Ho-Hd cattle under heat stress. Conversely, the relative expression levels of anti-apoptotic proteins, including Bcl-2, HSP 27, and HSP70, in ear cells derived from SCNT-Yo-Hd cattle are significantly higher than those in ear cells derived from SCNT-Ho-Hd cattle under heat stress [19].

Theoretically, generating reconstructed embryos using ooplasm from Y and nuclei from H is a feasible approach to generate H with improved heat tolerance that can be inherited by their offspring [20]. However, SCNT embryos containing nuclei from somatic cells may encounter incomplete genetic reprogramming that leads to reduced embryo survival rates, pregnancy rates, and postnatal fetal survival rates [21]. In contrast, ST oocytes are produced by transferring karyoplasts derived from H oocytes into recipient ooplasm from Y, followed by fertilization with H sperm, to produce ST embryos, so the issue of incomplete genetic reprogramming does not exist. Our group has successfully produced cloned cattle by transferring H nuclei into Y oocytes and fertilizing them with H sperm, resulting in ST cattle (ST-Yo-Hn) with 100% Taiwan yellow cattle cytoplasm and 100% Holstein cow nuclei [22].

To compare the differentially expressed molecular profiles of ear fibroblasts from ST-Yo-Hn, purebred H, and purebred Y in response to heat shock, various mRNA and proteins, including anti-apoptotic and pro-apoptotic factors and factors related to oxidation, phosphorylation, and antioxidants, were examined under different conditions, with or without heat shock. The results of the present study are fundamental to understanding the molecules involved in mediating the heat tolerance of bovine breeds.

## 2. Materials and Methods

### 2.1. Animals

Nine cattle were utilized in the present study, including three ST-Yo-Hn, three purebred Y, and three H. The ST-Yo-Hn were produced using ooplasm derived from Y and donor nuclei from H [22]. All experimental protocols regarding animal use were approved by the Institutional Animal Care and Use Committee (IACUC; approval number: NPUST-IACUC-110-137) of National Pingtung University of Science and Technology, Taiwan.

### 2.2. Preparation of Ear Fibroblasts and Culture

Ear fibroblasts were prepared as previously described by Kesorn et al. [20]. Ear tissue samples were collected from all nine cattle, which were approximately one year old. The samples were then carefully cut into uniform pieces measuring 3 mm^2^ each and cultured in Dulbecco’s modified Eagle’s medium (DMEM, Gibco, Grand Island, NY, USA) containing 1% (*v*:*v*) penicillin/streptomycin (Gibco, 15140-122) and 10% (*v*:*v*) fetal bovine serum (FBS, Gibco, 10270-106). The tissue samples were cultured in a CO_2_ incubator under specific conditions of 38.5 °C, 5% CO_2_, 95% air, and saturated humidity. The ear fibroblasts were cultured continuously until reaching passage 3. At each passage, cells were cryopreserved using DMEM containing 90% (*v*:*v*) FBS and 10% (*v*:*v*) dimethyl sulfoxide (DMSO; Sigma, D-5879, St. Louis, MO, USA) in liquid nitrogen until further analysis.

### 2.3. Ear Fibroblast Heat Shock Treatment

A heat shock treatment was applied to the ear fibroblasts following a protocol outlined by Lee et al. [19]. Fibroblast cells from passage 3 were stored in liquid nitrogen and thawed in a water bath at 37 °C for 25 s. The cells were then cultured in DMEM supplemented with 1% penicillin and streptomycin and 10% FBS and placed in an incubator at 38.5 °C with 5% CO_2_ and 95% air for 24 h (passage 4). Following this, the cells were trypsinized and resuspended at a concentration of 1 × 10^6^ cells/mL in DMEM supplemented with 10% FBS for further cultivation. The non-heat shock (control) group was incubated under standard conditions of 38.5 °C, 5% CO_2_, 95% air, and saturated humidity. In contrast, the heat-treated group was subjected to the same conditions except the temperature was raised to 42 °C while the CO_2_ was increased to 7%. Finally, after 12 h of treatment, cells of the fifth passage were collected for mRNA and protein extraction.

### 2.4. Evaluation of Thermotolerance of Ear Fibroblasts

#### 2.4.1. Analysis of Protein Expression Types

##### Sample Preparation

Ear fibroblast cells from each treatment were washed twice with DPBS and subsequently treated with 50 μL of a radioimmunoprecipitation assay (RIPA) lysis buffer (Millipore, Temecula, CA, USA) for a period of 15 min while kept on ice. A proteinase inhibitor cocktail (Roche, Mannheim, Germany) was then added to terminate the reaction. The resulting mixture was later centrifuged at 20,000× *g* at 4 °C for 15 min, and the supernatant was collected to determine the total protein concentration in the cell lysate using a Protein Assay Kit II (Bio-Rad, Hercules, CA, USA). The protein concentration of each sample was adjusted to 50 μg/μL, and all samples were then frozen at −80 °C in preparation for Western blotting and an Enzyme-Linked Immunosorbent Assay (ELISA).

##### Analysis of Protein Expression Types Utilizing Liquid Chromatography–Mass Spectrometry (LC-MS/MS)

Protein samples were sent to a biotechnology company (Biotools Company, New Taipei City, Taiwan) for a series of analytical procedures that included in-gel digestion, LC-MS/MS analysis, and protein identification. The in-gel digestion procedure involved destaining, followed by reduction using 10 mM dithiothreitol (DTT, Merck, Darmstadt, Germany) at 56 °C for 45 min. Cysteine blocking was then performed using 55 mM iodoacetamide (IAM, Sigma, St. Louis, MO, USA) at 25 °C for 30 min. The resulting samples were digested with sequencing-grade modified porcine trypsin (Promega, Madison, WI, USA) at 37 °C for 16 h. The peptides were subsequently extracted from the gel, dried using vacuum centrifugation, and stored at −80 °C until further use. 

Further analysis involved dilution of digested peptides in HPLC buffer A (0.1% formic acid) and loading onto a reverse-phase column (Zorbax 300SB-C18, 0.3 × 5 mm; Agilent Technologies, Wilmington, DE, USA). The desalted peptides were then separated on a homemade column (HydroRP 2.5 µm, 75 μm I.D. × 20 cm with a 15 μm tip) using a multi-step gradient of HPLC buffer B (99.9% acetonitrile/0.1% formic acid) for 70 min with a flow rate of 0.3 μL/min. The LC apparatus was coupled with a 2D linear ion trap mass spectrometer (Orbitrap Elite ETD; Thermo Fisher, San Jose, CA, USA), which was operated using Xcalibur 2.2 software (Thermo Fisher, San Jose, CA, USA). Full-scan MS was performed in the Orbitrap over a range of 400 to 2000 Da with a resolution of 120,000 at an *m*/*z* of 400. Internal calibration was performed using the ion signal of [Si(CH3)2O]6H+ at an *m*/*z* of 536.165365 as a lock mass. The experiment included 20 data-dependent MS/MS scan events followed by 1 MS scan for the 20 most abundant precursor ions in the preview MS scan. The *m*/*z* values selected for MS/MS were dynamically excluded for 40 s with a relative mass window of 15 ppm. The electrospray voltage was set to 2.0 kV, and the capillary temperature was set to 200 °C. MS and MS/MS automatic gain control were set to 1000 ms (full scan) and 200 ms (MS/MS) or 3 × 10^6^ ions (full scan) and 3 × 10^3^ ions (MS/MS) for the maximum accumulated time or ions, respectively. 

Proteome Discoverer software (version 1.4, Thermo Fisher Scientific, Waltham, MA, USA) was utilized to analyze the data. The MS/MS spectra were searched in the UniProt and NCBI (RefSeq) databases using the Mascot search engine (Matrix Science, London, UK; version 2.5). Peptide identification was allowed mass tolerances of 10 ppm for intact peptide masses and 0.5 Da for CID fragment ions with allowance for two missed cleavages from trypsin digestion. Variable modifications included oxidized methionine and acetyl (protein N-terminal), while carbamidomethyl (cysteine) was considered a static modification. Peptide–spectrum matches (PSMs) were filtered based on high confidence and Mascot search engine rank 1 for peptide identification, ensuring an overall false discovery rate below 0.01. Proteins with a single peptide hit were subsequently excluded from the analysis [23,24].

##### Selection of Candidate Genes and Proteins

The LC-MS/MS analysis was used to identify proteins that were differentially expressed in response to heat stress in cattle. Peptide–spectrum match (PSM) values were obtained for all proteins in the LC-MS/MS analysis data from each treatment group (H: *n* = 3; Y: *n* = 3; ST-Yo-Hn: *n* = 3, with or without heat shock). Based on the PSM values, different expression profiles were established for the heat-shocked groups and their corresponding control groups. Subsequently, the correlation among the three cattle groups (H, Y, and ST-Yo-Hn) was assessed for proteins exhibiting differential expression in response to heat shock. Candidate genes and proteins were selected for further analysis based on their association with apoptosis and antioxidant functions. Apoptosis-related proteins such as G protein subunit alpha 14 (GNA14), Cysteine-rich with EGF-like domains 2 (CRELD2), and Protein kinase C theta (PRKCQ), as well as antioxidant-related proteins including Superoxide dismutase 1 (SOD1), Catalase (CAT), 6-phosphogluconolactonase (PGLS), and Glutathione peroxidase 1 (GPX1), were identified and selected for analysis. Proteins associated with apoptosis (Caspase-3, Caspase-8, and Caspase-9) and those involved in oxidative phosphorylation (Complex-Ⅰ, Complex-II, Complex-III, and Complex-IV) were also analyzed. Relative mRNA expression analysis was performed for *GPX1*, *CRELD2*, and *PRKCQ* due to the unavailability of suitable antibodies, while relative protein expression analysis was conducted for the other 11 proteins.

#### 2.4.2. Analysis of the Relative Gene Expression Levels Utilizing RT-PCR

mRNA was extracted from the fibroblast cells of Holstein cows using a Gene-Spin total RNA purification kit (Bio-protech, PT-RNA-MS-50, Taipei, Taiwan). The extracted mRNA was subjected to reverse transcription using an MMLV Reverse Transcription Kit (Bio-protech, PT-RT-KIT-100, Taipei, Taiwan) to synthesize cDNA. The cDNA was then analyzed for the expression of candidate genes using the SYBR Green I-labeled RT-PCR method. The primer sequences for the candidate genes are provided in Table 1. The RT-PCR reaction was carried out using a specially prepared 10-fold-concentrated reaction mix and the cDNA obtained earlier. Each PCR reaction mixture was subjected to multiple cycles under specific conditions. Subsequently, a melting curve analysis was performed to determine the melting temperature (Tm value) of the main PCR product (target gene) and the primer dimers in each reaction tube.

The data obtained from the RT-PCR reactions were processed using Light Cycler 480 Software v1.5.0 and input into Light Cycler Relative Quantification Software. The Comparative Quantification Method (2Cp Method) was then applied for the relative quantification analysis of the candidate genes. In this analysis, the expression levels of all candidate genes were compared with those of the ear fibroblast cells from the untreated (control) group of Holstein cows, which were considered to have a relative gene expression level of 1 [25]. Finally, the expression levels of the candidate genes were analyzed in terms of the internal control (GAPDH gene expression) for relative quantification correction. The relative expression levels of all candidate genes were compared in each analysis with those of the ear fibroblast cells from the untreated group (control group) of Holstein cows, which were considered to have a relative gene expression level of 1.

#### 2.4.3. Detection of Relative Expression of Proteins

##### Western Blotting

The protein expression of various genes, including GNA14, Complex-I, Complex-II, Complex-III, Complex-IV, SOD1, CAT, and PGLS, in ear fibroblast cells was determined using Western blotting analysis. The experimental procedure involved loading 50 μg of protein extracted from ear fibroblast cells in each treatment group into SDS-PAGE gel (12%) and subjecting it to electrophoresis at 80 V for 2 h. The proteins were then transferred to a PVDF membrane (Amersham Biosciences, GE Healthcare Europe GmbH, Diegem, Belgium) and blocked with 2% BSA at 37 °C for 1 h. The primary antibodies used in this experiment included anti-GNA14, anti-Complex I, anti-Complex II, anti-Complex III, anti-Complex IV, anti-SOD1, anti-CAT, anti-PGLS, and anti-β-actin antibodies. The PVDF membrane was incubated with the primary antibodies and washed. Thereafter, it was incubated with horseradish peroxidase (HRP) secondary antibodies. The relative expression levels of various proteins were determined using an ECL kit (Millipore) for chemiluminescence analysis. Further, images were obtained using an illumination imaging system (UVP, Upland, CA, USA) and subjected to densitometric analysis for the quantification of each protein using a Bio-imaging camera system. The quantification of the various proteins was normalized to the β-actin content in each treatment group as an internal control. Finally, the relative expression levels of all candidate proteins were compared in each analysis with those of the ear fibroblast cells from the untreated group (control group) of Holstein cows, which were considered to have a relative protein expression level of 1 [19].

##### Enzyme-Linked Immunosorbent Assay (ELISA)

An ELISA kit was used to analyze the relative expression levels of caspase-3, -8, and -9 in ear fibroblast cells from the control and heat-treated groups, following a standard procedure. Briefly, 96-well plates were coated with unlabeled capturing antibodies against caspase-3, -8, and -9 (each at 1:2000, Santa Cruz Biotechnology, Heidelberg, Germany) and kept at room temperature for 2 h. Subsequently, DPBS–Tween-20 and a DPBS buffer were used for washing and blocking, respectively. Specimens were then added to each well and allowed to react for 2 h at room temperature. The detecting antibodies against the respective caspases were then added, followed by HRP-labeled secondary antibodies (1:5000, Bioss, Woburn, MA, USA) incubated at room temperature for 2 h. A colorimetric reaction was initiated by adding an orthophenylene diamine substrate. The relative expression levels of the different caspases were compared in each analysis with those of the ear fibroblast cells from the untreated (control) group of Holstein cows, which were considered to have a relative protein expression level of 1 [19].

### 2.5. Experimental Designs

#### 2.5.1. Evaluation of the Protein Profiles of Heat-Shocked Bovine Ear Fibroblasts from Cattle with Different Cytoplasmic Origins

The effects of the heat shock treatment on ear fibroblast cells derived from the 3 cattle groups (H, Y, and ST-Yo-Hn) were investigated. Ear fibroblasts from the 3 cattle groups were subjected to 2 treatments: the control treatment (38.5 °C) and heat shock (42 °C) for 12 h. LC-MS/MS was employed to analyze the protein differences among the 6 combinations (3 cattle groups × 2 incubation temperatures), and peptide–spectrum match (PSM) values were used to compare the estimated protein expression levels between the ear fibroblasts with and without heat shock for each cattle group. The primary objective was to identify proteins that were differentially expressed in response to the heat shock treatment in each cattle group, which were further subjected to correlation analysis.

#### 2.5.2. Effects of Heat Shock Treatment on the Expression of Apoptosis-Related Factors in Bovine Ear Fibroblasts with Different Cytoplasmic Origins

Ear fibroblasts from H, Y, and ST-To-Hn cattle were incubated at either 38.5 °C (control) or 42 °C (heat shock) for 12 h. Afterwards, the levels of pro-apoptotic factors (Caspase-3, Caspase-8, and Caspase-9) and anti-apoptotic factors (*CRELD2* and *PRKCQ* genes) in each treatment were analyzed by ELISA (pro-apoptotic factors) and RT-PCR (anti-apoptotic factors). GNA14 was analyzed by Western blotting.

#### 2.5.3. Effects of Heat Shock Treatment on the Expression of Electron Transport Chain-Related Factors in Bovine Ear Fibroblasts with Different Cytoplasmic Origins

Ear fibroblasts derived from H, Y, and ST-To-Hn cattle were incubated at either 38.5 °C (control) or 42 °C (heat shock) for 12 h. Samples from all experimental treatments were analyzed using Western blotting (Complex-Ⅰ, Complex-II, Complex-III, Complex-IV, SOD1, CAT, and PGLS) and RT-PCR (GPx1).

### 2.6. Statistical Analysis

All experimental data were analyzed using general linear models (GLMs) in SAS (version 9.1). Mean values among groups were compared using Duncan’s new multiple ranges test, where a *p* value lower than 0.05 (*p* < 0.05) was considered significant.

## 3. Results

### 3.1. Evaluation of the Protein Profiles of Heat-Shocked Bovine Ear Fibroblasts from Cattle with Different Cytoplasmic Origins

**Figure 1:**
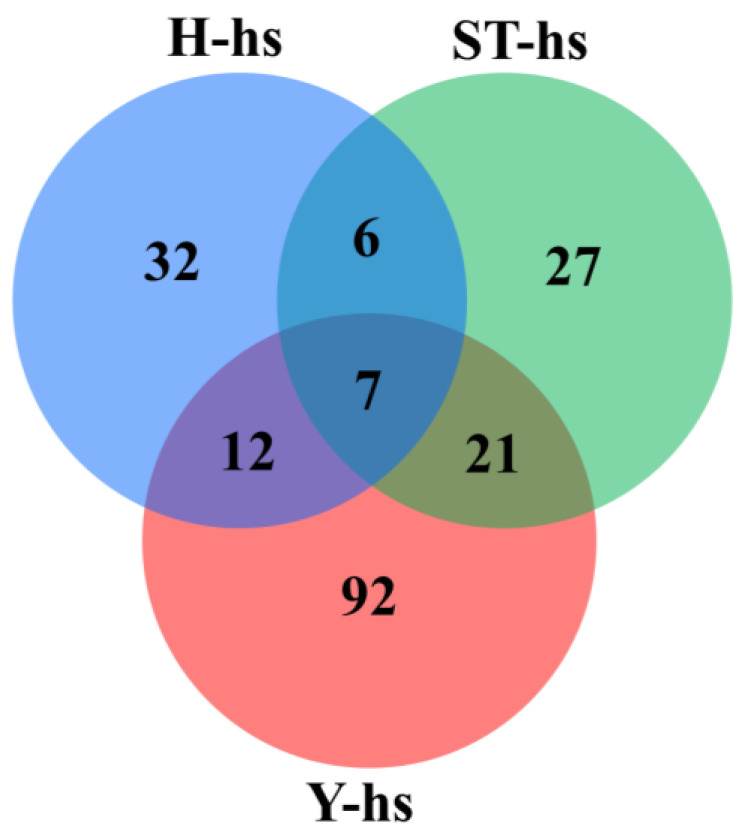
Differentially expressed protein profiles in ear fibroblasts derived from cattle with different cytoplasmic origins in response to heat shock (42 °C for 12 h). H: ear fibroblasts derived from Holstein cattle; Y: ear fibroblasts derived from Taiwan yellow cattle; ST: ear fibroblasts derived from ST cattle produced by embryos reconstructed with Taiwan yellow cattle ooplasm and Holstein nuclei (ST-Hd-Yo). Each treatment was repeated three times.

A comparison of the protein expression in ear fibroblast cells from cattle subjected to heat treatments is illustrated in Figure 1. The results indicate that the H-hs, Y-hs, and ST-Yo-Hn-hs groups had 32, 92, and 27 differentially expressed protein species, respectively. A correlation assessment of the differentially expressed protein species among the three cattle breeds under heat stress indicated a shared subset of seven differentially expressed protein species. The H-hs and ST-Yo-Hn-hs groups had 6 overlapping proteins, while the H-hs and Y-hs groups had 12 and the Y-hs and ST-Yo-Hn-hs groups had 21. Since the cytoplasm in ST-Yo-Hn cattle originates from Y cattle, target proteins were screened from the 21 differentially expressed proteins that were shared by the ST-Yo-Hn-hs and Y-hs groups (Figure 1). The selected target proteins included GNA14, CRELD2, and PRKCQ, which are associated with apoptosis, as well as SOD1, CAT, PGLS, and GPX1, which are related to antioxidation.

### 3.2. Effects of Heat Shock Treatment on the Expression of Apoptosis-Related Factors in Bovine Ear Fibroblasts with Different Cytoplasmic Origins

**Figure 2:**
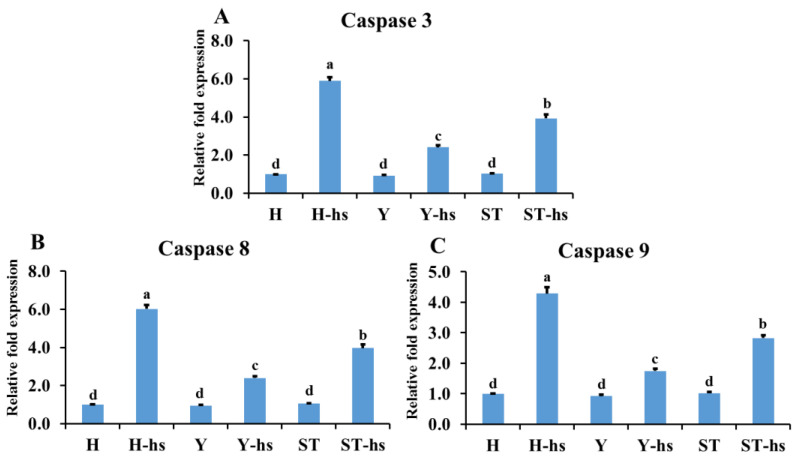
The relative expression of Caspase-3 (**A**), -8 (**B**), and -9 (**C**) proteins in ear fibroblasts derived from cattle with different cytoplasmic origins in response to heat shock (42 °C for 12 h). All values were calculated using the relative expression compared to non-heat-shocked ear fibroblasts from the same individual. H: ear fibroblasts derived from Holstein cattle; Y: ear fibroblasts derived from Taiwan yellow cattle; ST: ear fibroblasts derived from ST cattle produced by embryos reconstructed with Taiwan yellow cattle ooplasm and Holstein nuclei (ST-Hd-Yo). ^a, b, c, d^ Values without the same superscripts are significantly different (*p* < 0.05). Each treatment was repeated three times.

**Figure 3:**
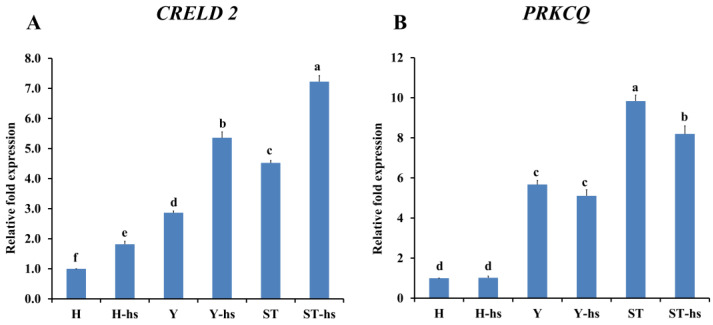
Relative mRNA abundance of *CRELD2* (**A**) and *PRKCQ* (**B**) genes in ear fibroblasts derived from cattle with different cytoplasmic origins in response to heat shock (42 °C for 12 h). All values were calculated using the relative expression compared to non-heat-shocked ear fibroblasts from the same individual. H: ear fibroblasts derived from Holstein cattle; Y: ear fibroblasts derived from Taiwan yellow cattle; ST: ear fibroblasts derived from ST cattle produced by embryos reconstructed with Taiwan yellow cattle ooplasm and Holstein nuclei (ST-Hd-Yo). ^a, b, c, d, e, f^ Values without the same superscripts are significantly different (*p* < 0.05). Each treatment was repeated three times.

**Figure 4:**
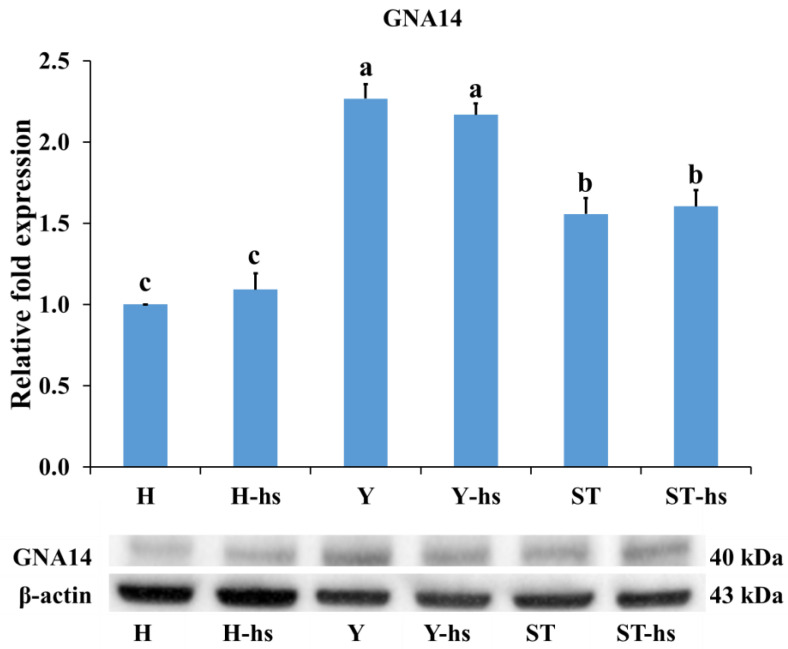
The relative expression of GNA14 protein in ear fibroblasts derived from cattle with different cytoplasmic sources after heat shock (42 °C for 12 h). Western blot analysis of GNA14 from matching samples (original Western blot figures are presented in Figure S4). β-actin served as an internal control, and all values were calculated using the relative expression compared to non-heat-shocked ear fibroblasts from the same individual. H: ear fibroblasts derived from Holstein cattle; Y: ear fibroblasts derived from Taiwan yellow cattle; ST: ear fibroblasts derived from ST cattle produced by embryos reconstructed with Taiwan yellow cattle ooplasm and Holstein nuclei (ST-Hd-Yo). ^a, b, c^ Values without the same superscripts are significantly different (*p* < 0.05). Each treatment was repeated three times.

The findings revealed that pro-apoptotic factors such as Caspase-3 (Figure 2A), Caspase-8 (Figure 2B), and Caspase-9 (Figure 2C), had the lowest protein expression in the Y-hs group, while the H-hs group had the highest expression. Furthermore, the ST-Yo-Hn-hs group demonstrated lower expression levels than the H-hs group (*p* < 0.05) (Figure 2). In terms of anti-apoptotic factors, both the ST-Yo-Hn-hs and Y-hs groups had significantly (*p* < 0.05) higher mRNA expression levels of *CRELD2* (Figure 3A) and *PRKCO* (Figure 3B) than the H-hs group. GNA14 (Figure 4) had the highest protein expression level in the Y-hs group (*p* < 0.05), followed by the ST-Yo-Hn-hs group, while the H-hs group had the lowest expression (*p* < 0.05).

### 3.3. Effects of Heat Shock Treatment on the Expression of Electron Transport Chain-Related Factors in Bovine Ear Fibroblasts with Different Cytoplasmic Origins

**Figure 5:**
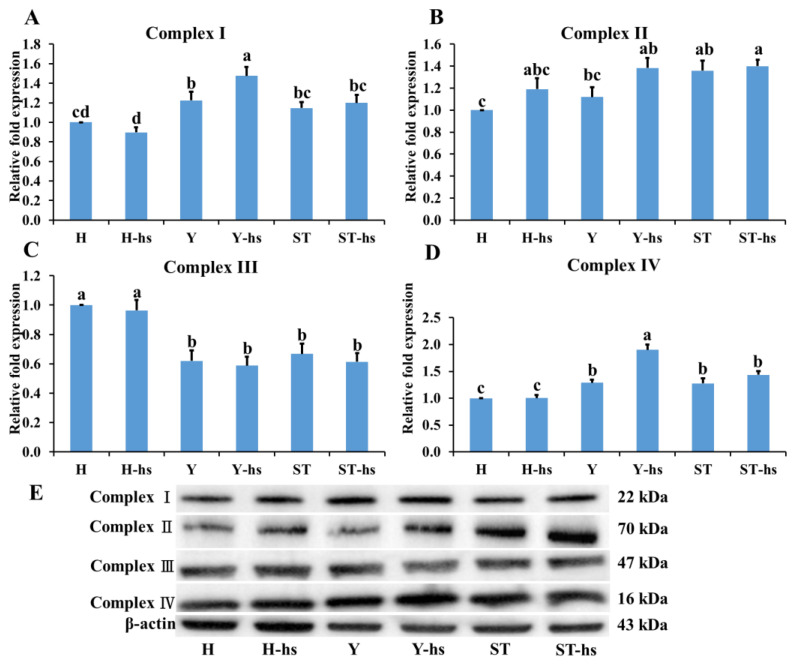
The relative expression of Complex-I (**A**), -II (**B**), -III (**C**), and -IV (**D**) proteins in ear fibroblasts derived from cattle with different cytoplasmic origins in response to heat shock (42 °C for 12 h). (**E**) Western blot analysis of Complex-I (**A**), -II (**B**), -III (**C**), and -IV (**D**) from matching samples (original Western blot figures are presented in Figure S5). β-actin served as an internal control, and all values were calculated using the relative expression compared to non-heat-shocked ear fibroblasts from the same individual. H: ear fibroblasts derived from Holstein cattle; Y: ear fibroblasts derived from Taiwan yellow cattle; ST: ear fibroblasts derived from ST cattle produced by embryos reconstructed with Taiwan yellow cattle ooplasm and Holstein nuclei (ST-Hd-Yo). ^a, b, c, d^ Values without the same superscripts are significantly different (*p* < 0.05). Each treatment was repeated three times.

**Figure 6:**
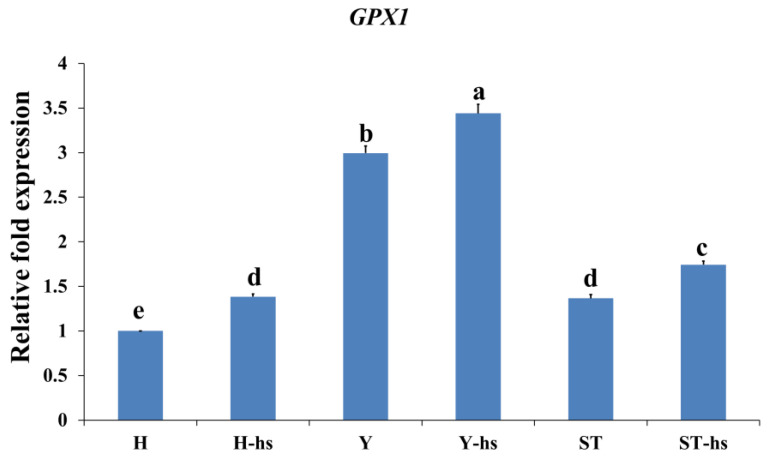
Relative mRNA abundance of *GPX1* gene in ear fibroblasts derived from cattle with different cytoplasmic origins in response to heat shock (42 °C for 12 h). All values were calculated using the relative expression compared to non-heat-shocked ear cells from the same individual. H: ear fibroblasts derived from Holstein cattle; Y: ear fibroblasts derived from Taiwan yellow cattle; ST: ear fibroblasts derived from ST cattle produced by embryos reconstructed with Taiwan yellow cattle ooplasm and Holstein nuclei (ST-Hd-Yo). ^a, b, c, d, e^ Values without the same superscripts are significantly different (*p* < 0.05). Each treatment was repeated three times.

**Figure 7:**
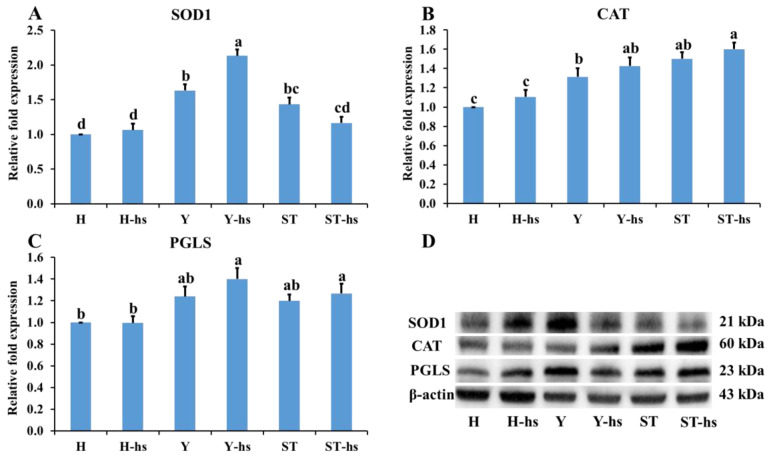
The relative expression of SOD1 (**A**), CAT (**B**), and PGLS (**C**) proteins in ear fibroblasts derived from cattle with different cytoplasmic origins in response to heat shock (42 °C for 12 h). (**D**) Western blot analysis of SOD1 (**A**), CAT (**B**), and PGLS (**C**) from matching samples (original Western blot figures are presented in Figure S7). β-actin served as an internal control, and all values were calculated using the relative expression compared to non-heat-shocked ear fibroblasts from the same individual. H: ear fibroblasts derived from Holstein cattle; Y: ear fibroblasts derived from Taiwan yellow cattle; ST: ear fibroblasts derived from ST cattle produced by embryos reconstructed with Taiwan yellow cattle ooplasm and Holstein nuclei (ST-Hd-Yo). ^a, b, c, d^ Values without the same superscripts are significantly different (*p* < 0.05). Each treatment was repeated three times.

The levels of electron transport chain-related factors in ear cells with different cytoplasmic sources under heat stress were also determined. The results showed that the Y-hs group had significantly higher relative protein expression levels of Complex-I (Figure 5A) and Complex-IV (Figure 5D) compared to the ST-Yo-Hn-hs group (*p* < 0.05) and that the ST-Yo-Hn-hs group had significantly higher expression levels than the H-hs group (*p* < 0.05). Complex-II (Figure 5B), in contrast, showed no significant differences among the three groups (*p* > 0.05), while Complex-III (Figure 5C) was significantly higher in the H-hs group compared to the Y-hs and ST-Yo-Hn-hs groups (*p* < 0.05). Additionally, the Y-hs group had significantly higher relative mRNA expression levels of *GPX1* (Figure 6) compared to the ST-Yo-Hn-hs group (*p* < 0.05), while the ST-Yo-Hn-hs group had significantly higher *GPX1* expression levels than the H-hs group (*p* < 0.05). SOD1 (Figure 7A) showed significantly higher relative protein expression levels in the Y-hs group compared to the H-hs and ST-Yo-Hn-hs groups (*p* < 0.05), while CAT (Figure 7B) and PGLS (Figure 7C) had no significant differences in their relative expression levels between the Y-hs and ST-Yo-Hn-hs groups (*p* > 0.05) but were significantly lower in the H-hs group (*p* < 0.05).

## 4. Discussion

Global warming poses a significant threat to the dairy industry in tropical and subtropical regions, especially during the hot season. Heat stress caused by rising temperatures can lead to hormonal imbalances in dairy cows, which result in lower fertility rates, higher miscarriage rates, and decreases in milk production. To mitigate the impact of heat stress, one strategy is to crossbreed a native heat-tolerant breed (*Bos indicus*, BI) with a heat-sensitive breed with high milk production (*Bos taurus*, BT). For example, native breeds can be crossbred with Holstein cattle [13,14]. However, hybrid cows generally show lower heat tolerance than local breeds [15]. Recent research has indicated that introducing heat-tolerant cattle cytoplasm into cattle cells can improve thermotolerance in heat-sensitive cattle (*Bos Taurus*, BT) [16,17,19]. 

Taiwan, which contains tropical and subtropical regions, has a native heat-tolerant cow breed known as Taiwan yellow cattle (Y). In our research, the somatic cell nuclear transfer (SCNT) technique was used to transfer donor nuclei (d) from Holstein (H) cattle to ooplasm (o) from Taiwan yellow cattle (Y) (SCNT-Yo-Hd). The findings suggest that this process leads to significantly lower expression of pro-apoptotic factors and higher expression of anti-apoptotic factors in ear cells under heat stress [19,20]. Moreover, our research indicates that thermotolerance can be inherited maternally [20]. However, SCNT animals often have incomplete genetic reprogramming, leading to developmental abnormalities [21]. In contrast, spindle transfer (ST) technology avoids such reprogramming issues by using nuclei from oocytes that are then fertilized with sperm. Our group successfully produced ST cattle using nuclei derived from oocytes and sperm from H with ooplasm from Y (ST-Yo-Hn) [22]. However, the molecular mechanisms underlying thermotolerance in ST-Yo-Hn cattle remain unclear. Therefore, this study assessed the superior heat tolerance of ST-Yo-Hn cattle by comparing the molecular profiles of ear cells derived from both ST-Yo-Hn cattle and purebred H under heat stress.

Heat stress that affects the fates of cells, whether toward apoptosis or survival, is regulated by the interplay between pro-apoptotic and anti-apoptotic factors [26,27]. In addition, heat stress impairs mitochondrial biogenesis, which includes fission and fusion processes that strive to maintain normal energy production and antioxidant activity, and decreases reactive oxygen species (ROS)-induced cellular damage [28,29,30,31]. Mitochondrial dysfunction leads to the production of ROS, which are regulated by numerous genes and proteins [32,33,34,35]. This study primarily analyzed genes and proteins related to heat stress, including pro-apoptotic factors such as Caspase-3, -8, and -9, whose activation leads to apoptosis [36,37]. The gene expression of pro-apoptotic factors in the ST-Yo-Hn-derived ear cells, including Caspase-3 (Figure 2A), -8 (Figure 2B) and -9 (Figure 2C), was significantly lower than in the H-hs group under heat stress. These results align with previous research showing that ear cells derived from SCNT-Yo-Hd cattle (with Y-derived cytoplasm) exhibit significantly lower expression of Caspase-3, -8, and -9 under heat stress compared to SCNT-Ho-Hd (with H-derived cytoplasm) and pure Holstein cattle [19,20]. This suggests that somatic cells of ST-Yo-Hn cattle indeed exhibit lower expression of pro-apoptotic factors under heat stress conditions.

Although exploration of anti-apoptotic factors in cytoplasm originating from different cattle breeds has not been conducted, previous research has shown that ear cells derived from SCNT-Yo-Hd cattle exhibit significantly higher expression of anti-apoptotic factors, such as HSP-70 and Bcl-2, under heat stress compared to SCNT-Ho-Hd cattle [19,20]. These findings suggest that the higher expression patterns of the anti-apoptotic factors *CRELD2*, *PRKCQ*, and *GNA14* in ST-Yo-Hn cattle are similar to the higher expression of HSP-70 and Bcl-2 in SCNT-Yo-Hd cattle, indicating that Y cytoplasm may contribute to the expression of these anti-apoptotic factors [19,20].

The process of energy production in mitochondria involves the use of oxidative phosphorylation, which generates ROS, such as Complex-I, Complex-II, Complex-III, and Complex-IV [38,39,40]. It has been observed that ear cells from SCNT-Yo-Hd cattle, which possess Y cytoplasm, exhibit significantly elevated levels of Complex-I, Complex-III, and Complex-IV expression under heat stress conditions compared to SCNT-Ho-Hd cattle [41]. These findings are consistent with the present research, in which the ST-Yo-Hn-hs group had higher expression of Complex-I and Complex-IV protein in comparison with the H-hs group (Figure 5A,D). Likewise, research conducted on mouse myocytes and C2C12 myotubes subjected to heat stress (40 °C for 1 h) and then cultured at 37 °C for 23 h demonstrated increased heat tolerance and higher levels of Complex-I, -II, -III, and -IV protein expression [31]. Furthermore, studies have found that heat shock protein-72 (HSP-72) expression increased in rat myocardial cells under heat stress treatment while reducing cytochrome C release from mitochondrial membranes. This led to enhanced activity of mitochondrial complex proteins [42]. Under stress conditions, the mitochondrial electron transport chain triggers antioxidant systems to maintain mitochondrial function and ensure proper cellular energy production [40].

Antioxidant enzymes such as GPX1, SOD1, CAT, and PGLS play a crucial role in reducing oxidative stress and removing ROS to ameliorate cellular damage [43,44]. GPX1, SOD1, and CAT convert superoxide radicals (O_2_•−) produced in the mitochondrial respiratory chain into O_2_ and H_2_O, while PGLS inhibits the expression of ROS in human hepatocellular carcinoma cells, thereby inducing apoptosis [43,44]. A previous study showed that dermal fibroblasts from heat-tolerant cattle breeds, such as Tharparkar cattle, show significantly lower oxidative stress levels after heat stress compared to heat-sensitive breeds like Karan-Fries cattle [45]. This could be due to the fact that heat-sensitive breeds have inferior antioxidant capabilities [46,47], while cells from heat-tolerant breeds exhibit significantly higher anti-oxidative capacities after heat stress [48,49]. In a previous study, it was observed that the relative expression of GPX and CAT proteins in ear fibroblasts of SCNT-Yo-Hd cattle was significantly higher than that in SCNT-Ho-Hd cattle under heat stress. 

Similarly, in this study, the expression of antioxidant genes such as *GPX1* (Figure 6) and proteins like CAT (Figure 7B) and PGLS (Figure 7C) was considerably higher in the ST-Yo-Hn-hs group compared to the H-hs group. These results suggest that ST-Yo-Hn cattle, which are produced with cytoplasm originating from Y, might possess enhanced efficiency in energy production and enhanced antioxidant capabilities under heat stress. Therefore, these enhanced capabilities could effectively eliminate the ROS generated due to heat stress, resulting in superior heat tolerance abilities [30,31,41].

In summary, our results demonstrate that ear fibroblasts from ST-Yo-Hn cattle are significantly more thermotolerant than those from H. Since reductions in milk production under heat stress have significant negative impacts on the dairy industry in tropical areas, this approach could be used to produce bovine breeds with high milk production, such as H, with improved heat tolerance so that reductions in milk yield under heat stress can be mitigated. However, further research is required to investigate the actual milk production performance of these ST-Yo-Hn cattle.

## 5. Conclusions

The findings of this study suggest that somatic cells (ear cells) from ST-Yo-Hn cattle, with a combination of nuclei from Holstein cattle and cytoplasm from Taiwan yellow cattle, exhibit thermotolerance under heat stress. These findings are supported by significant decreases in pro-apoptotic factors and a notable increase in gene expression related to anti-apoptotic factors, oxidative phosphorylation, and antioxidant activity. These molecular mechanisms demonstrate the superior heat tolerance of ST-Yo-Hn cattle compared to H cattle. This heat tolerance is likely due to the cytoplasm from the heat-tolerant cattle breed, which is consistent with prior research indicating that cytoplasm from heat-tolerant cattle breeds can enhance the heat tolerance of cell nuclei from heat-sensitive cattle breeds.

## Figures and Tables

**Table 1:** Primer sets used for the qPCR analysis of the target genes.

Genes	Primer Sequence (5′-3′)	Size (bp)
*GPX1*	F: CAGATGAATGACCTGCAGCG R: GACGTACTTCAGGCAATTCAGGAT	126
*CRELD2*	F: GTGCTCCGACTGCATGGAC R: CGCAGTCCCTGTTGGTGG	116
*PRKCQ*	F: AGGATGAAGAGGAGCTTTTCCA R: CGCTTCTCAGGCTCTCTTACG	118
*GAPDH*	F: AGTGGACATCGTCGCCATC R: CGTTCTCTGCCTTGACTGTGC	113

## Data Availability

Data are available only upon agreement with the breeding organization and should be requested directly from the authors.

## Supplementary Materials

The following are available online at https://www.mdpi.com/article/10.3390/ani14091371/s1, Figure S4. Whole western blot and densitometry reading/intensity ratio of GNA14 and β-actin. The relative fold expression of GNA14 protein in ear fibroblasts derived from different cattle groups after heat-shock (42 °C, 12 h). Figure S5. Whole western blot and densitometry reading/intensity ratio of Complex-I, -II, -III, -IV and β-actin. The relative fold expression of Complex-I, -II, -III and -IV proteins in ear fibroblasts derived from different cattle groups after heat-shock (42 °C, 12 h). Figure S7. Whole western blot and densitometry reading/intensity ratio of SOD1, CAT, PGLS and β-actin. The relative fold expression of SOD1, CAT and PGLS proteins in ear fibroblasts derived from different cattle groups after heat-shock (42 °C, 12 h).
