# Supplementary material for: Identification of Molecular Profile of Ear Fibroblasts Derived from Spindle-Transferred Holstein Cattle with Ooplasts from Taiwan Yellow Cattle under Heat Stress
Source: Animals (Basel). 2024 May 2;14(9):1371. doi: 10.3390/ani14091371 (PMC11083940; doi:10.3390/ani14091371)
Supplement: Supplementary file 1 [file animals-14-01371-s001.zip › animals-2984077-supplementary.pdf]

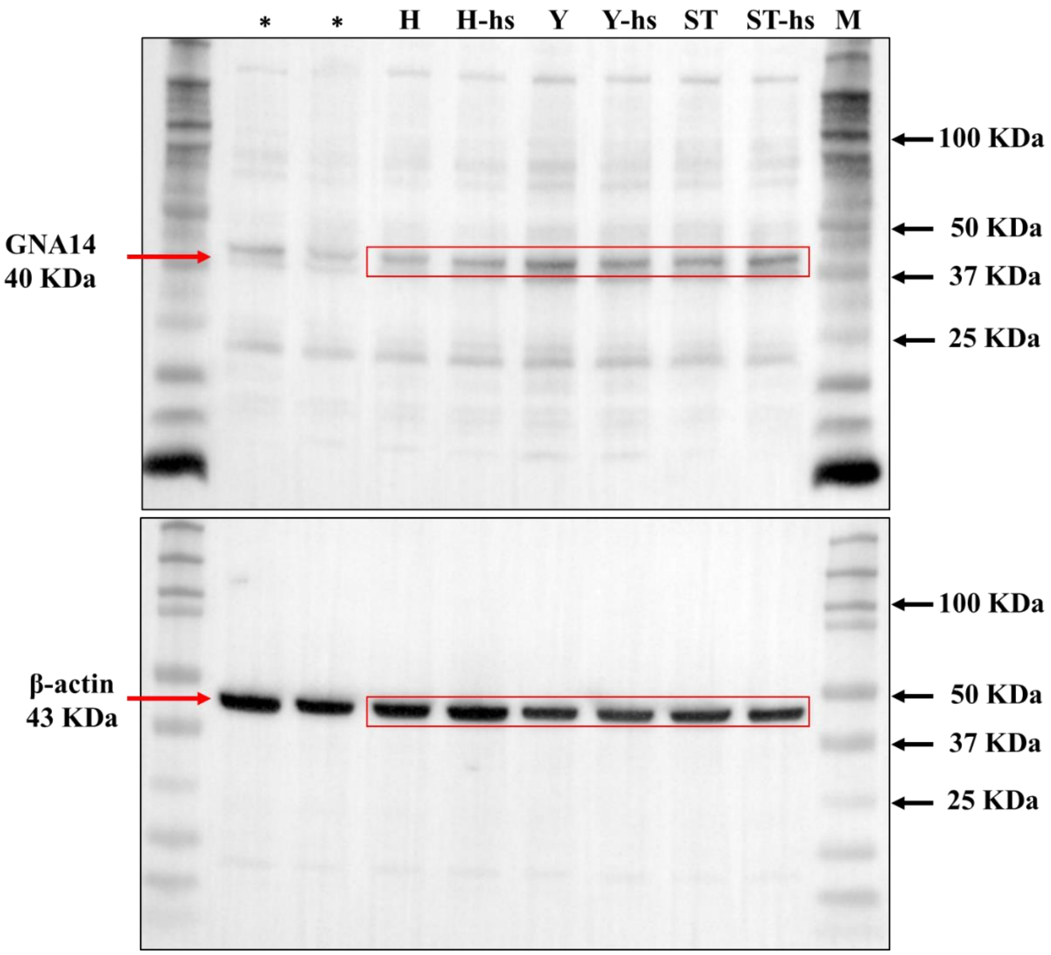

	H	H-hs	Y	Y-hs	ST	ST-hs
GNA14/β-actin	0.243	0.246	0.564	0.520	0.350	0.391

Figure S4. Whole western blot and densitometry reading/intensity ratio of GNA14 and β-actin. The relative fold expression of GNA14 protein in ear fibroblasts derived from different cattle groups after heat-shock (42°C, 12 h). β-actin serves as the internal control. H: ear fibroblasts derived from Holstein cattle; Y: ear fibroblasts derived from Taiwan yellow cattle; ST: ear fibroblasts derived from ST cattle produced by the embryos reconstructed with Taiwan yellow cattle ooplasm and Holstein nucleus (ST-Hd-Yo). * **The left two lanes are samples from other experiment.**

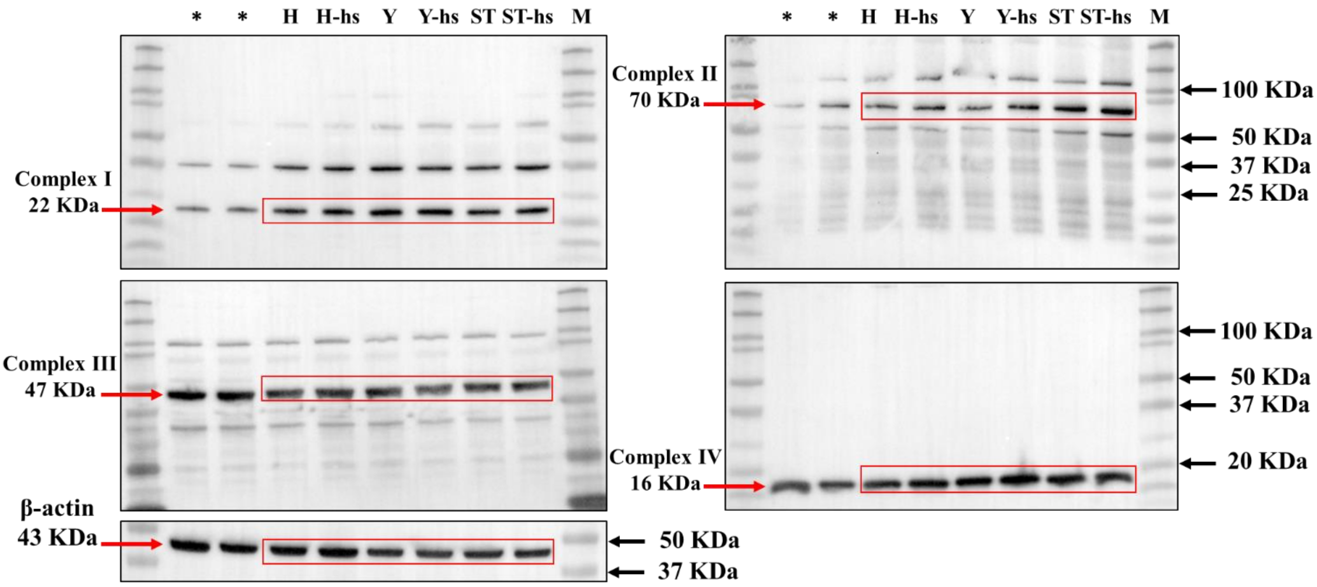

	H	H-hs	Y	Y-hs	ST	ST-hs
Complex Ⅰ/β-actin	0.704	0.627	0.869	0.938	0.868	0.848
Complex Ⅱ/β-actin	0.465	0.661	0.641	0.767	0.848	0.867
Complex Ⅲ/β-actin	0.929	0.980	0.981	0.991	0.997	1.082
Complex Ⅳ/β-actin	0.912	0.980	1.209	1.735	1.025	1.220

Figure S5. Whole western blot and densitometry reading/intensity ratio of Complex-I, -Ⅱ, -Ⅲ, -Ⅳ and β-actin. The relative fold expression of Complex-I, -Ⅱ, -Ⅲ and -Ⅳ proteins in ear fibroblasts derived from different cattle groups after heat-shock (42°C, 12 h). β-actin serves as the internal control. H: ear fibroblasts derived from Holstein cattle; Y: ear fibroblasts derived from Taiwan yellow cattle; ST: ear fibroblasts derived from ST cattle produced by the embryos reconstructed with Taiwan yellow cattle ooplasm and Holstein nucleus (ST-Hd-Yo). ***The left two lanes are samples from other experiment.**

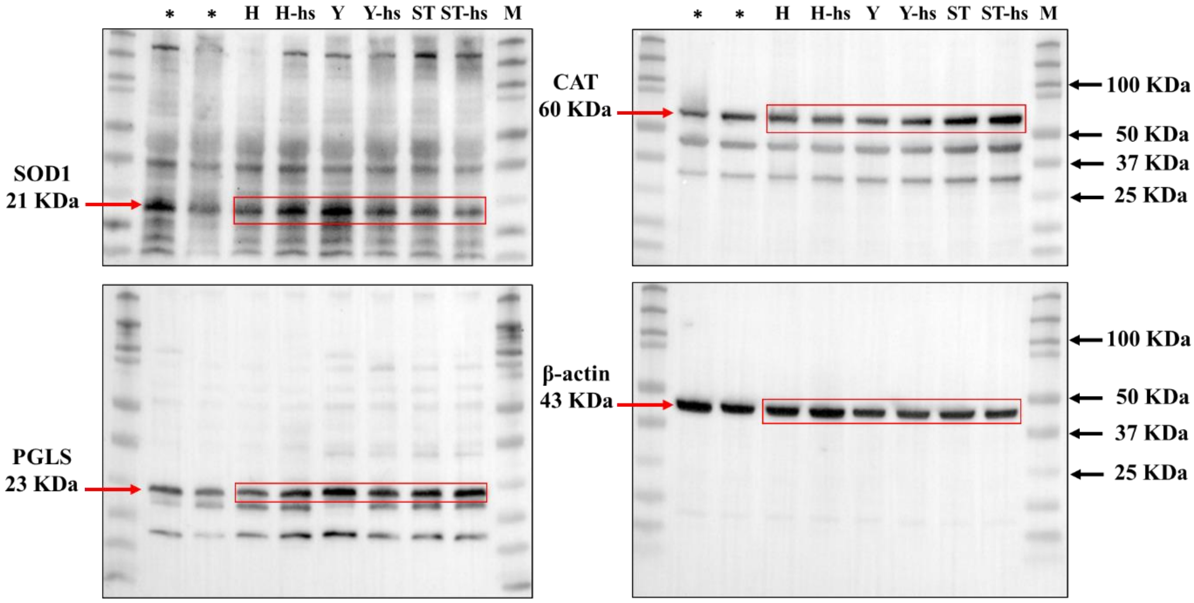

	H	H-hs	Y	Y-hs	ST	ST-hs
SOD1/β-tubulin	0.583	0.728	1.247	0.967	0.786	0.701
CAT/β-tubulin	0.535	0.598	0.812	0.863	0.991	1.231
PGLS/β-tubulin	0.462	0.665	1.168	0.895	0.925	1.086

Figure S7. Whole western blot and densitometry reading/intensity ratio of SOD1, CAT, PGLS and β-actin. The relative fold expression of SOD1, CAT and PGLS proteins in ear fibroblasts derived from different cattle groups after heat-shock (42°C, 12 h). β-actin serves as the internal control. H: ear fibroblasts derived from Holstein cattle; Y: ear fibroblasts derived from Taiwan yellow cattle; ST: ear fibroblasts derived from ST cattle produced by the embryos reconstructed with Taiwan yellow cattle ooplasm and Holstein nucleus (ST-Hd-Yo). *** The left two lanes are samples from other experiment.**
